# Supplementary material for: Phenotypic plasticity in the mandibular morphology of Japanese macaques: captive–wild comparison
Source: R Soc Open Sci. 2019 Jul 10;6(7):181382. doi: 10.1098/rsos.181382 (PMC6689643; doi:10.1098/rsos.181382)
Supplement: supplementary materials [file rsos181382supp1.pdf]

Table S1. Multivariate regression: test for the effects of age and generation on PC4 and shape score.

	Estimate	Std. Error	t value	Pr(>|t|)
PC4				
Intercept	-0.02	0.29	-0.06	0.95
size	-1.1E-03	0.06	-0.02	0.98
sex	-2.2E-03	0.01	-0.28	0.78
age class	0.01	0.01	1.96	0.06
age (in years)	6.1E-04	4.4E-04	1.36	0.18
generation	-1.6E-03	2.4E-03	-0.65	0.52
Shape score				
Intercept	0.13	0.25	0.54	0.59
size	-0.02	0.05	-0.46	0.65
sex	0.01	0.01	1.29	0.20
age class	-0.01	4.9E-03	-1.08	0.29
age (in years)	-6.9E-05	3.8E-04	-0.18	0.86
generation	-3.2E-03	2.0E-03	-1.54	0.13

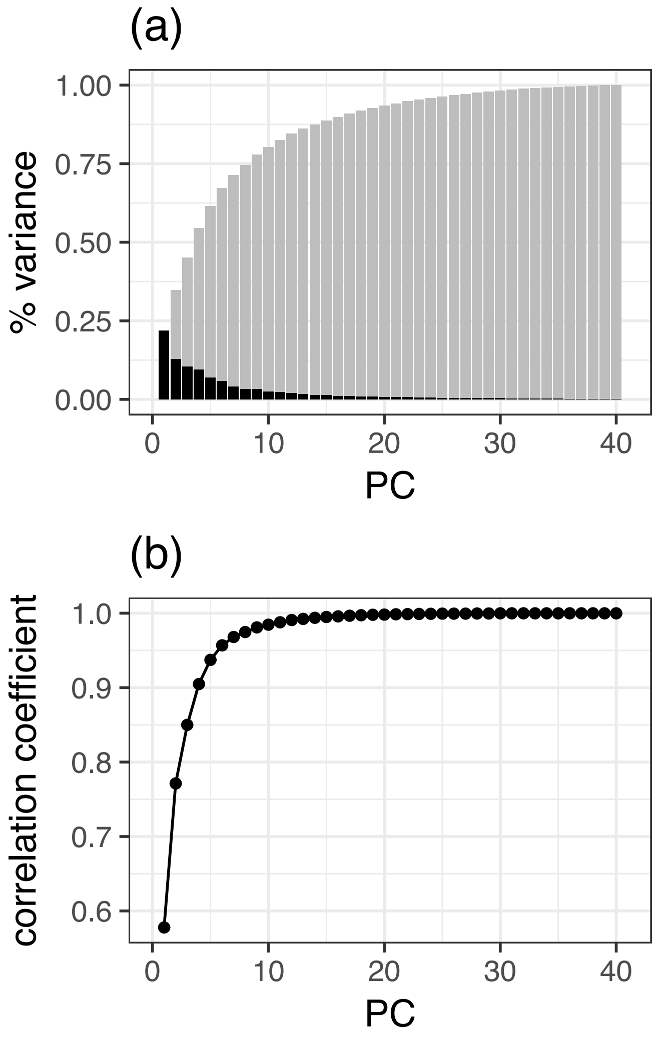

Figure S1. The summary of principal component analysis. (a) The variance (black) and cumulative variance (gray) of principal components. (b) The correlation between the matrices of principal component scores and Procrustes distances.

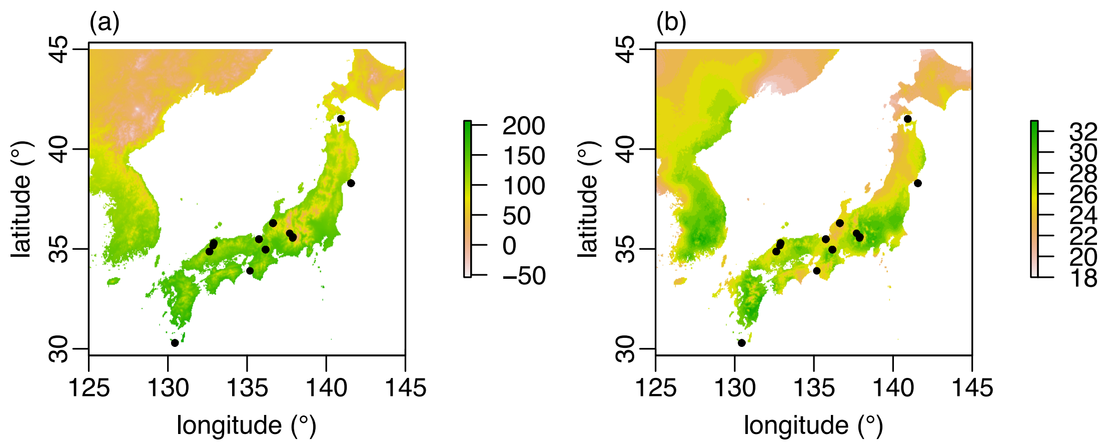

Figure S2. The ecogeographical variables from WorldClim database. (a) Annual mean temperature (°C × 10). (b) Annual precipitation (mm).

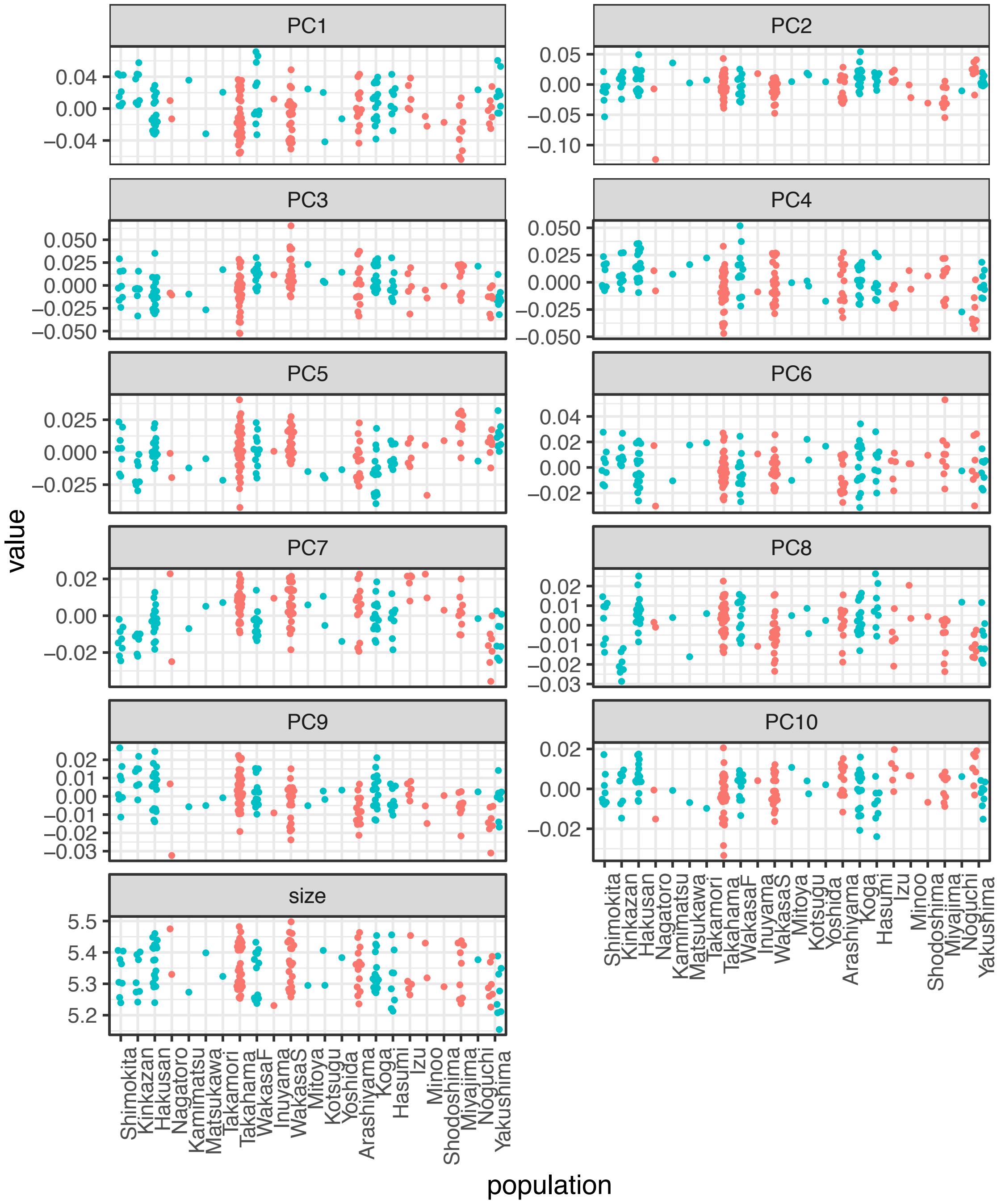

Figure S3. Plots of PC scores and size. Red indicates captive individuals and blue, wild individuals.

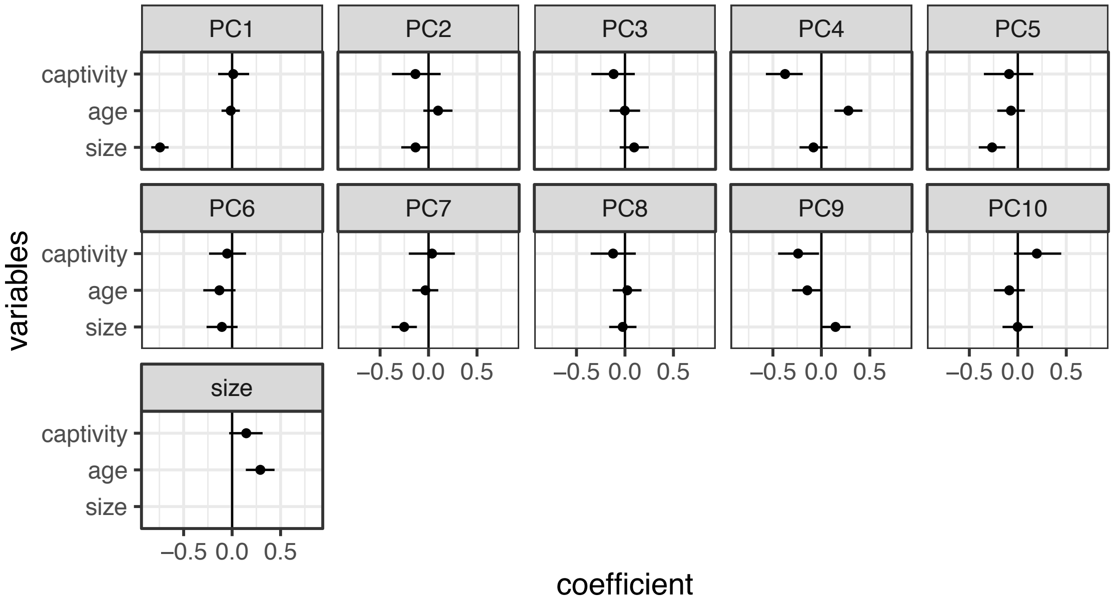

Figure S4. Posterior distributions of regression coefficients in the test of captivity when removing sex from explanatory factors. Points are means and lines represent 95% credible intervals.

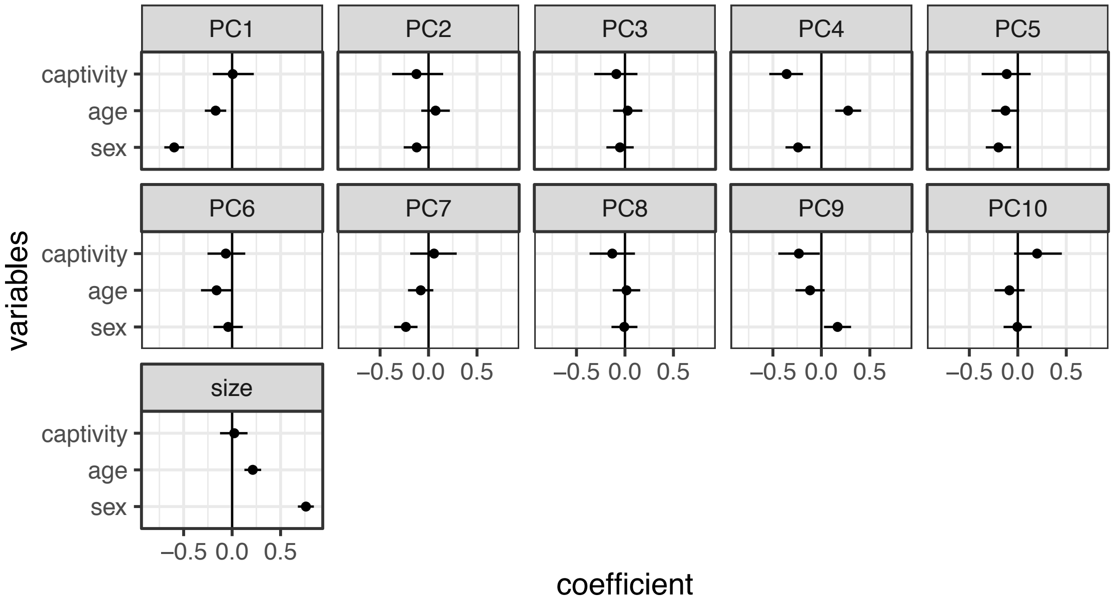

Figure S5. Posterior distributions of regression coefficients in the test of captivity when removing size from explanatory factors. Points are means and lines represent 95% credible intervals.

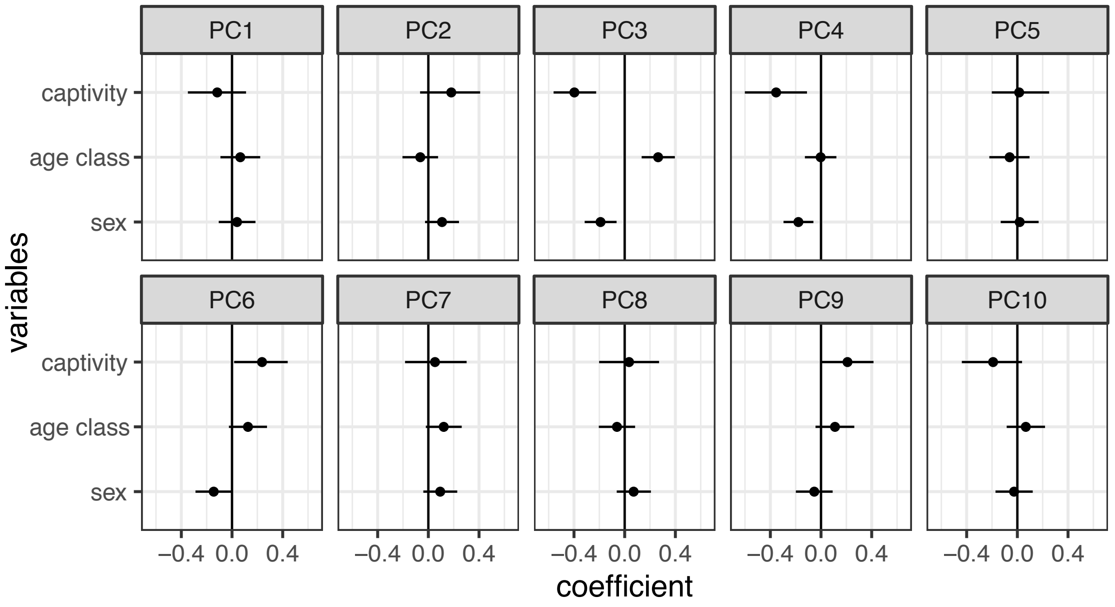

Figure S6. Posterior distributions of regression coefficients in the test of captivity for the PCs of size-adjusted shape data. Points are means and lines represent 95% credible intervals. Captivity has significant effect for PC2 (−0.40 [95% CI −0.56 to −0.23]), PC3 (−0.35 [−0.60 to −0.11]), and PC6 (0.24 [0.02—0.44]). The WAIC of full model is much smaller than the reduced model, in which captivity is excluded from among the explanatory variables (Δ WAIC = 15.39 ± 10.4).

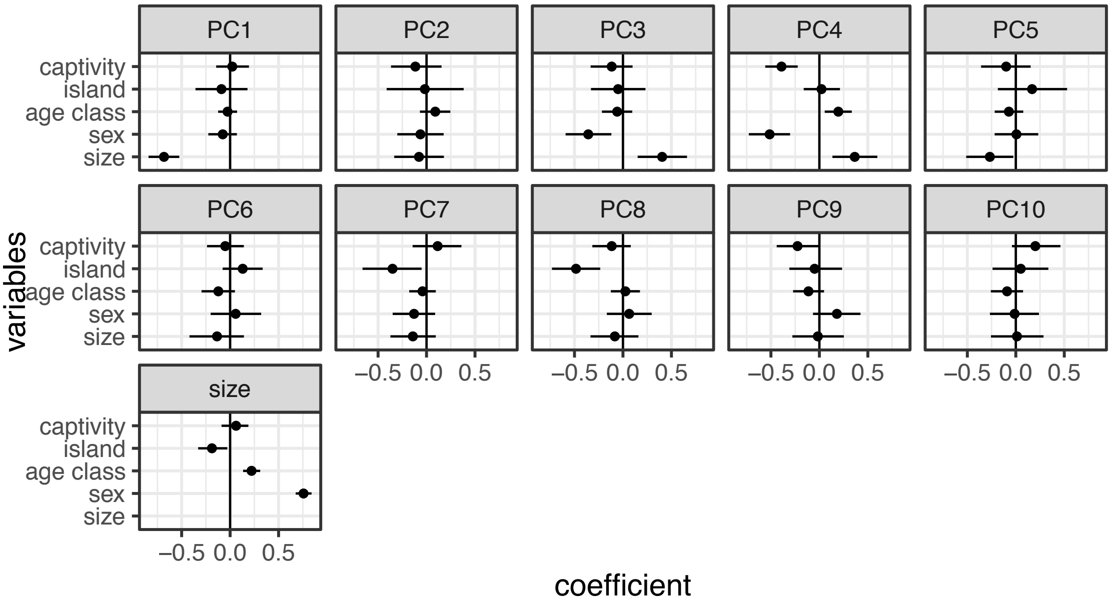

Figure S7. Posterior distributions of regression coefficients in the test of captivity when including island for explanatory factor. Points are means and lines represent 95% credible intervals. Captivity has significant effect for PC4 (−0.39 [95% CI −0.56 to −0.22]). The WAIC of full model is smaller than the reduced model, in which captivity is excluded from among the explanatory variables (Δ WAIC = 6.46 ± 10.33).

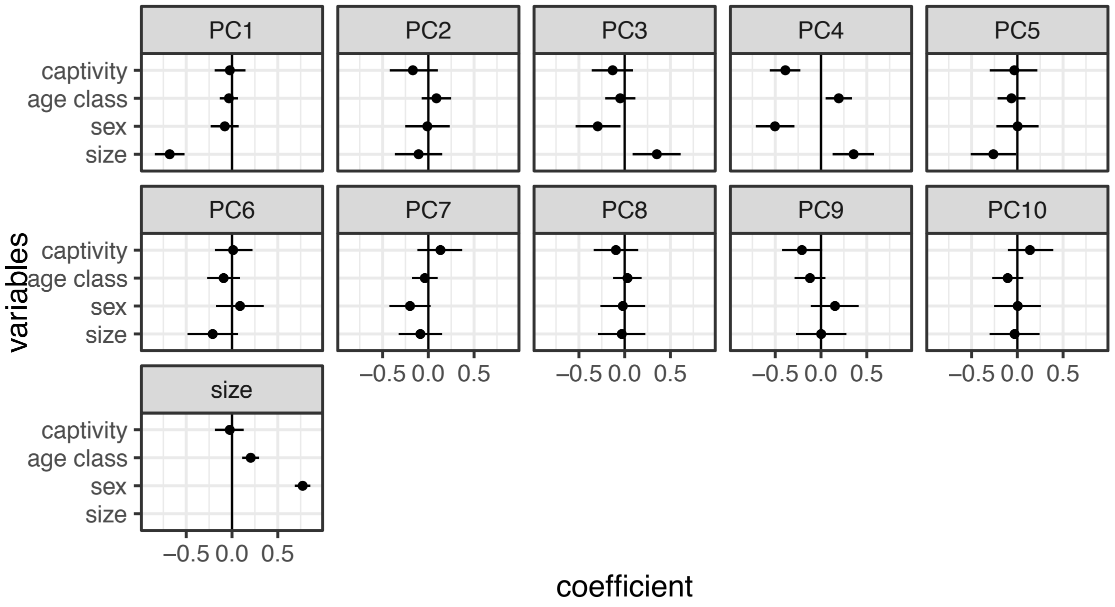

Figure S8. Posterior distributions of regression coefficients in the test of captivity when using the subset of samples that excludes more than 20-years-old captive individuals. Points are means and lines represent 95% credible intervals. Captivity has significant effect for PC4 (−0.39 [95% CI −0.56 to −0.23]). The WAIC of full model is smaller than the reduced model, in which captivity is excluded from among the explanatory variables (ΔWAIC = 5.74 ± 10.53).

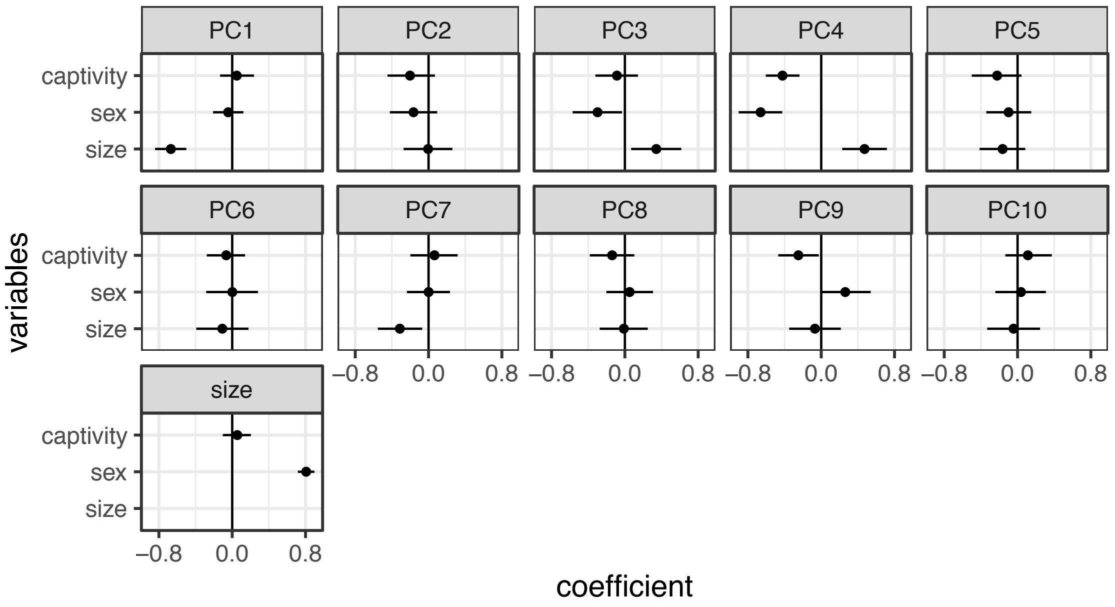

Figure S9. Posterior distributions of regression coefficients in the test of captivity when using the subset of samples that includes only adult individuals. Points are means and lines represent 95% credible intervals. Captivity has significant effect for PC4 (−0.42 [95% CI −0.61 to −0.24]). The WAIC of full model is smaller than the reduced model, in which captivity is excluded from among the explanatory variables (Δ WAIC = 7.64 ± 10.14).

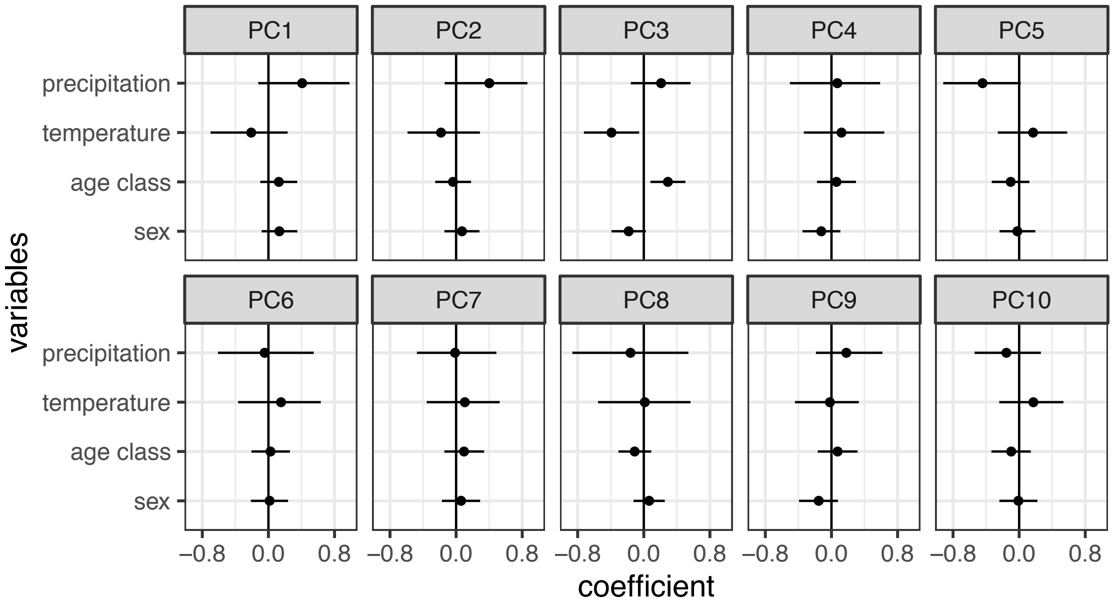

Figure S10. Posterior distributions of regression coefficients in the test of ecogeographical factors for the PCs of size-adjusted shape data. Points are means and lines represent 95% credible intervals. Temperature has significant effect for PC3 (−0.39 [95% CI −0.72 to −0.06]). However, the best model suggested by WAIC is the reduced one, in which both temperature and precipitation are excluded from among the explanatory variables [ΔWAIC (to the worst full model) = 9.92 ± 6.77].

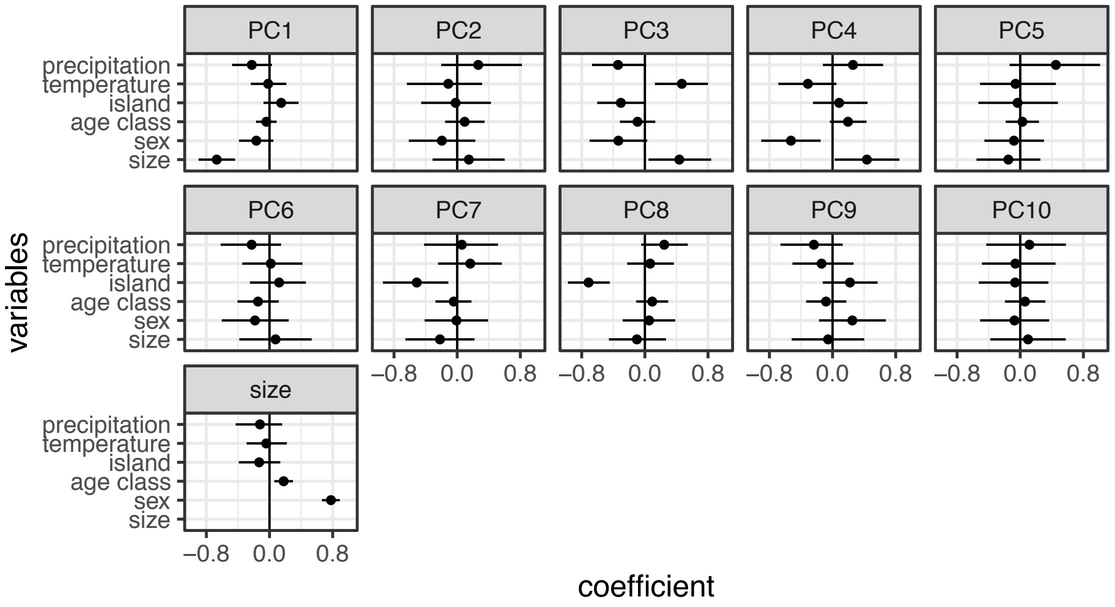

Figure S11. Posterior distributions of regression coefficients in the test of ecogeographical factors when including island for explanatory factor. Points are means and lines represent 95% credible intervals. Temperature has significant effect for PC3 (0.47 [95% CI 0.13 to 0.80]). The best model suggested by WAIC is the one, in which precipitation is excluded from among the explanatory variables [Δ WAIC (to the reduced model, in which both temperature and precipitation are excluded) = 3.92 ± 6.74].
